# Assisting decision-making on age of neutering for German Short/Wirehaired Pointer, Mastiff, Newfoundland, Rhodesian Ridgeback, Siberian Husky: associated joint disorders, cancers, and urinary incontinence

**DOI:** 10.3389/fvets.2024.1322276

**Published:** 2024-04-12

**Authors:** Lynette Arnason Hart, Abigail Paige Thigpen, Benjamin L. Hart, Neil H. Willits, Maya Lee, Miya M. Babchuk, Jenna Lee, Megan Ho, Sara T. Clarkson, Juliann W. Chou

**Affiliations:** ^1^School of Veterinary Medicine, University of California, Davis, Davis, CA, United States; ^2^Department of Statistics, College of Letters and Science, University of California, Davis, Davis, CA, United States

**Keywords:** hip dysplasia, elbow dysplasia, cranial cruciate ligament tear, lymphosarcoma, mast cell tumor, hemangiosarcoma, osteosarcoma, 40 dog breeds

## Abstract

Spaying female and castrating male dogs, hereinafter referred to as neutering, is a US convention for the first year in the dog's life. Research on 35 breeds of dogs revealed that early neutering increases risks of joint disorders, such as hip dysplasia (HD), elbow dysplasia (ED), or cranial cruciate ligament (CCL) tear, or cancers, such as lymphosarcoma (LSA), mast cell tumor (MCT), hemangiosarcoma (has), or osteosarcoma (OSA), for some breeds. Joint disorder risks are heightened for some larger breeds and for mixed-breed dogs weighing more than 20 kg. Some breeds had elevated risks for cancers. Several other research teams have reported health complications associated with neutering. The study goal includes using the same methodology for data collection and analyses as in the study on 35 breeds for five additional dog breeds weighing at least 20 kg. The breeds were: German Short/Wirehaired Pointer, Mastiff, Newfoundland, Rhodesian Ridgeback, and Siberian Husky. Major differences among breeds appeared in vulnerability to joint disorders and cancers with early neutering: male and female Pointer breeds had elevated joint disorders and increased cancers; male Mastiff breeds had increased CCL and LSA and females had non-significant elevated CCL risks; female Newfoundland breeds had heightened risks for joint disorders and males had non-significant elevated risks; female Ridgeback breeds had heightened MCT with very early neutering; and Siberian Huskies showed no significant effects on joint disorders or cancers, but female breeds showed a non-significant but elevated CCL. Updated guidelines cover 40 dog breeds. These results further emphasize the importance of personalized decisions regarding the neutering of dogs, considering the dog's breed, sex, and context.

## Introduction

In the 1980s−90s, the prevalence of neutering dogs increased in the United States, with concerns about overpopulation and hopes to reduce behavioral problems. Neutering refers to castration of male breeds, generally involving surgical removal of the testicles, or spaying of female breeds (ovariohysterectomy), including removal of both the uterus and ovaries. Alternatives that also prevent reproduction but are performed far less frequently include vasectomy of male breeds and tubal ligation of female breeds or removing the uterus and/or ovaries. For female breeds, laparoscopic methods are used in ovary-sparing hysterectomy and ovariectomy (1). Chemical/hormonal sterilants have also been administered to male and female dogs.

Surgical methods for neutering puppies became popular and assured the prevention of reproduction in dogs adopted from shelters. Neutering of male dogs was accelerated by studies showing a decrease in aggression toward family members in a minority of neutered dogs (2–4). However, in the 2000s, concerns grew about the behavioral and health impacts of neutering for some dogs, creating a complicated picture of the likely effects of neutering a specific dog at certain ages. Research has emphasized that several cancers (5–8) and joint disorders (9, 10) were more common in neutered than intact dogs, but there is a lack of information to inform veterinary practice.

For more breed, sex, and age-of-neutering information, patient records of Golden Retrievers were examined for risks of joint disorder, such as hip dysplasia (HD), elbow dysplasia (ED), or cranial cruciate ligament (CCL) tear, or cancer, such as lymphosarcoma (LSA), mast cell tumor (MCT), hemangiosarcoma (HSA), or osteosarcoma (OSA) (11). Age of neutering had major specific effects on risks for male and female dogs for the abovementioned three joint disorders and four cancers. Subsequently, 35 breeds (12) and 3 weight classes (13) of mixed breeds were examined for the roles of age of neutering on these health risks. Regarding joint disorders, some heavier dog breeds (over 20 kg) were at higher risk than lighter weight dogs. This pattern was also found with mixed-breed dogs, where those over 20 kg were more likely to have a significantly higher risk of joint disorders when neutered than intact. Other genetic diseases were found to have increased expression with neutering, especially when performed early and for female breeds (14). A further problem with neutering is that it results in lifelong extremely high secretion of the luteinizing hormone, having effects on receptors throughout the body, perhaps altering aggressive and fearful behaviors and various other disorders (15).

Dog owners' decisions on whether and when to neuter their dogs often are influenced by concerns about behavioral effects of neutering. Several recent studies have reported increased fear aggression in both male and female breeds having less exposure to gonadal hormones. Client surveys using the CBarq data have found that less lifetime exposure to gonadal hormones is associated with increased fearfulness and aggression in female (16) and male (17) dogs. A study with another database reported that dogs with longer exposure to gonadal hormones had fewer health issues and problem or nuisance behaviors (18).

With data published in 2020 on 35 breeds and mixed breeds of dogs highlighting the vulnerability of some large dogs to increased risks of joint disorders with early neutering (12, 13), and data of several more years available, hospital data were examined to identify any additional breeds of large dogs with sufficient data for similar analyses as previously used. This study reports results of five additional large breeds of male and female dogs that had a sufficient case load for analyses, all weighing more than 20 kg: German Wire/Shorthaired Pointer, Mastiff, Newfoundland, Rhodesian Ridgeback, and Siberian Husky. Some evidence already highlights the vulnerability of the five breeds to these conditions. Orthopedic Foundation for Animals (OFA) presents summary reports for abnormal hips and elbows: German Shorthaired Pointer−3.8% and 1.2%; Mastiff−20.5% and 15.7%; Newfoundland−25.9% and 23.6%; Rhodesian Ridgeback−4.4% and 6.5%; Siberian Husky−0.3% and 0.1% (19), respectively. These are data combined for intact male and female dogs, assessing the dogs for breeding. A comprehensive study of HD and CCL in male and female dogs by Witsberger et al. reported rates of the two disorders as follows: German Shorthaired Pointer−3% and 3%; Newfoundland−17% and 9%, the highest scores of all breeds examined; Siberian Husky−2% and 2% (10), respectively. In general, this study also reported heightened risks of both HD and CCL for neutered male dogs. A study of dogs <2 years of age with CCL, compared with age-matched controls, found that Mastiff and Newfoundland were two of the nine breeds at an increased risk for CCL (20). Neutering caused increased risks for both male and female dogs. An Egyptian study reported that 23% of Siberian Huskies had HD (21): figures were very different from those of the OFA and Witsberger's group. For various types of Mastiffs, cancer was the most common cause of mortality for 47% of dogs: OSA, 50% of cancer cases; LSA, 14%; HSA, 7% (22). In the US, in 2002, an available but unpublished paper on Rhodesian Ridgebacks from a breeder citing data by C. Roethal reported rates for MCT of 3.5% and HD of 2.2%; in Australia, in 2006, the rates for both conditions were very low: 0.2% (23). The unpublished paper mentioned that the breeding of Ridgebacks in the U.S. has reduced the HD incidence from 11.8% for dogs born before 1980 to 3.4% for dogs born in 2003/2004. Scores on a dog breed such as those from the OFA are derived from intact dogs that are being considered for breeding, representing a different pool than dogs in the general population of that breed.

This project sought to clarify the effects of age at neutering for these five breeds with joint diseases and cancers for dogs at the University of California Veterinary Medical Teaching Hospital. The methods were consistent with those in the earlier studies assessing diagnoses of three joint disorders and four cancers, as well as urinary incontinence (UI), pyometra (PYO), and mammary cancer (MC), with the status of intact or specific age of neutering. The specific objective was to identify the recommended ages for neutering of male and female dogs of these five breeds that did not increase the risks of a joint disorder or cancer. This information would inform veterinarians and their clients on how to optimize the age of neutering.

## Method

Patient records of the Veterinary Medical Teaching Hospital were reviewed for the five additional breeds weighing more than 20 kg with over 200 cases. The neuter status of each included animal was identified and specified as intact or neutered. The use of alternative procedures for neutering was not often observed or recorded. It is presumed that virtually all patients had conventional surgical neutering and that very few animals had less common alternative procedures, particularly any in which the dog retains the gonadal hormones.

### Ethics statement

The retrospective dataset was obtained from the Veterinary Medical Teaching Hospital (VMTH)'s hospital records. The campus policy allows the faculty of the University of California, Davis, to use the records for research. No animal care and use committee approval was required. Strict confidentiality for owners and their dogs was maintained.

### Study parameters, data collection, and presentation

Parameters, as in previous studies (11–13), examined occurrences of joint disorders: HD, CCL and ED and cancers: LSA, HSA, MCT, and OSA in both sexes. Mammary cancer (MC), pyometra (PYO), and urinary incontinence (UI) were examined in female dogs, and UI was examined in male breeds. The abovementioned diseases were examined for dogs left intact or neutered in the age periods: <6 months, 6–11 months, 1 year (12 to <24 months), or 2–8 years. The diseases were tracked through the dogs' last patient visit until 11 years of age.

The statistical evaluations, considering sex and different ages of neutering, required a large database. The study focused on proportional differences in disease occurrences between the neuter age groups and intact dogs of the same breed and sex.

The study period represented 21 years of data, from 1 January 2000 through 31 December 2020. The inclusion criteria were the date of birth, age at neutering (if neutered), and age of diagnosis or onset of clinical signs for diseases of interest. The term “early neutering” is sometimes used below to refer to neutering in the 1^st^ year, combining cases for both the <6 months and 6–11 months neuter periods. Female dogs were examined for MC, PYO, and UI and male dogs for UI. For all neutered dogs that developed a disease of interest, records were examined to confirm that the dog was neutered before the diagnosis or onset of signs of the disease. If the dog developed signs of the disease before neutering, the dog was considered intact for the analysis of that disease. However, for any disease that occurred after neutering, the dog was considered neutered for the analysis of that disease. For any disease of interest that occurred before 12 months of age, the dog was excluded from that disease analysis but included in analyses of other diseases. Thus, the number of cases for various diseases varied in the analyses for different disease occurrences.

When the age of neutering was not mentioned in the hospital records, telephone calls were made to the referring veterinarians to obtain the neutering dates or ages. When the age of neutering could not be obtained, the dog was excluded from the study, which was not an issue with intact dogs, so proportionately more intact cases appear in the final dataset for each breed than would be expected in the general population.

The criteria for disease diagnoses were the same as in previous studies on Golden Retrievers, 35 breeds, and mixed-breed dogs (11–13). A dog was considered as having a disease of interest if the diagnosis was made at the VMTH or by a referring veterinarian and later confirmed at the VMTH. For joint disorders (HD, ED, and/or CCL), dogs typically presented with signs of lameness, difficulty in moving, and/or joint pain. The diagnosis was confirmed by orthopedic examination, radiographic evidence, and/or surgery. UI was confirmed by clinical signs of abnormally frequent urination, urinalyses and exclusion of urinary tract infection, and/or other diseases. If a diagnosis was listed in the record as “suspected” based on some clinical signs but not confirmed, the case was excluded from the analysis for that specific disease, but the dog was included in other disease analyses.

### Statistical analyses

As in previous studies (11–13), survival analysis was used to test for differences with respect to the hazard of a disease in the neutered and intact groups while adjusting for the differences in time at risk for a disease. The groups were initially compared using a Kaplan–Meier life table analysis. *Post-hoc* comparisons among the subgroups were based on least squares means of the hazard within each subgroup. For comparisons where the Kaplan–Meier test showed significance at the *p*-value <0.05 level, both the log-rank and Wilcoxon tests were used for further analyses. Because joint disorders are expected to be seen at a similar risk throughout a dog's lifespan, regardless of age, the log-rank test was used for joint disorders. Conversely, cancers were examined with the Wilcoxon test since the risk of cancer is expected to be higher in older dogs. In contrast to previous studies (11–13) using this methodology, no asterisk was used in the joint disorder results to indicate significance seen only in the Wilcoxon test because all significant joint disorder results for the Wilcoxon test were also significant for the log-rank test. Similarly, no asterisk was used in the cancer results to indicate significance seen only in the log-rank test because all significant cancer results for the log-rank test were also significant for the Wilcoxon test. For all statistical tests, the two-tailed statistical level of significance was set at a *p*-value of <0.05 and reported as either a *p-*value of <0.05 or a *p*-value of <0.01. Each breed was analyzed separately, and there were no statistical comparisons between breeds. However, the overall findings with each breed allow for some general comparisons.

### Data presentation

For each of the five breeds in Appendix 1, the numbers of intact and neutered male and female dogs are given. Tables show percentages of dogs with each disease. Statistical analyses compared the occurrences of joint disorders and cancers between each neutering period and intact dogs. If the comparison was significant at either the *p*-value of <0.05 or p-value of <0.01 level, the data were bolded and the *p-*value was given. The detailed datasets are available online (Figshare, 10.6084/m9.figshare.24280054).

## Results

The breed-by-breed findings are presented in four formats. First, a short paragraph describing the occurrences of joint disorders and cancers for intact and neutered dogs for each breed is given below, with significant increases in disease types over intact dogs reported. Second, Table 1 presents guidelines reflecting research findings for all 40 breeds that have been studied, including the five breeds reported here. Third, the data-based findings, with statistical analyses for each breed, are reported in Appendix 1. Fourth, the raw data are available in Figshare.

**Table 1:** Suggested neutering age guidelines for 40 breeds by breed and sex.

**Suggested neutering age guidelines for 40 breeds**						
	Males			Females		
	No earlier than 6 months	No earlier than 12 months	No earlier than 24 months	No earlier than 6 months	No earlier than 12 months	No earlier than 24 months
Australian Cattle Dog	**X**			**X**		
Australian Shepherd	**X**			**X** ^*^		
Beagle		**X**		**X**		
Bernese Mountain Dog			**X**	**X**		
Border Collie		**X**			**X**	
Boston Terrier		**X**		**X**		
Boxer			**X**			**X**
Bulldog	**X**			**X**		
Cavalier King Charles Spaniel	**X**			**X**		
Chihuahua	**X**			**X**		
Cocker Spaniel	**X**					**X**
Collie	**X**				**X**	
Corgi	**X**			**X**		
Dachshund	**X**				**X**	
Doberman Pinscher	**Leave Intact**					**X** ^**^
English Springer Spaniel	**X**				**X**	
German Shepherd			**X**			**X**
German Short/Wirehaired Pointer		**X**			**X**	
Golden Retriever		**X**		**Leave Intact**		
Great Dane	**X**			**X**		
Irish Wolfhound			**X**	**X**		
Jack Russell Terrier	**X**			**X**		
Labrador Retriever	**X**				**X**	
Maltese	**X**			**X**		
Mastiff			**X**		**X**	
Miniature Schnauzer	**X**			**X**		
Newfoundland		**X**			**X**	
Pomeranian	**X**			**X**		
Poodle (Toy)	**X**			**X**		
Poodle (Miniature)		**X**		**X**		
Poodle (Standard)			**X**	**X**		
Pug	**X**			**X** ^*^		
Rhodesian Ridgeback	**X**			**X**		
Rottweiler		**X**		**X**		
Saint Bernard	**X**			**X**		
Shetland Sheepdog	**X**					**X** ^**^
Shih Tzu	**X**					**X**
Siberian Husky	**X**				**X**	
West Highland White Terrier	**X**			**X**		
Yorkshire Terrier	**X**			**X**		

Summary of spaying and neutering guidelines based on findings regarding joint disorders and cancers. ^*^Australian Shepherd and Pug female dogs had no statistically significantly elevated joint disorders or cancers with early neutering. Nonetheless, they are examples of breeds with more joint disorders and/or cancer cases among the early neutered animals, and these may have become significant in a larger dataset (12). ^**^This recommendation for Doberman Pinscher and Shetland Sheepdog females is based on their elevated risks of UI, not joint disorders or cancers (12).

### General findings

Data for these five large breeds of dogs provide cautionary information regarding neutering. The breeds were all rated in the top 50 by the American Kennel Club (AKC) in 2022 (23). Four breeds showed significantly elevated diagnoses with early neutering, and the fifth breed had some non-significant elevated scores. Brief summaries on results for each breed and suggested guidelines for neutering age are given below.

### German Short/Wirehaired Pointer

This dog enjoys increasing popularity, currently ranked #10 by AKC (24). These Pointers are used in hunting and excel in being highly trainable (25). The study population consisted of 150 intact male, 89 neutered male, 90 intact female, and 114 neutered female dogs, for a total sample of 443 cases. In intact male and female dogs, the occurrence of a joint disorder was 3% and 2%, respectively. CCL was slightly elevated for male dogs neutered before 1 year, and the occurrence of joint disorders was highly elevated for female breeds neutered before 6 months, with 38% having at least one joint disorder. Intact male and female breeds had a 6% and 1% likelihood of cancer, respectively. Male breeds neutered before 6 months had significantly elevated rates of MCT and HSA, both 8%, over intact male breeds with MCT (2%) and HSA (1%). Female breeds neutered before a year had a significantly increased risk for LSA, 11%. There were no cases of MC or pyometra among neutered female breeds, but intact female breeds had a 4% rate for each of these. Intact female breeds had no UI, but those neutered before 1 year had an insignificant increase to 6%; male dogs had almost no cases of UI. Given the increased rates of both joint disorders and cancers for both male and female dogs with early neutering, the suggested guideline is neutering no earlier than 12 months of age.

### Mastiff

This giant breed ranking #37 (24) weighs around 150 pounds. For a couple thousand years, Mastiffs guarded and protected households. They are above average in aggression toward other dogs and family and only average in trainability, so they are challenging to manage (25) but very favored by many people (22). The study population consisted of 148 intact male, 61 neutered male, 70 intact female, and 82 neutered female dogs, for a total sample of 361 cases. Intact male and female dogs had a 6% and 9% risk of at least one joint disorder, respectively. Neutering male dogs before 2 years resulted in a significantly elevated risk for a joint disorder, mainly due to CCL; neutering before 1 year was a 21% risk, and neutering at 1 year of age was a 15% risk. Although not significant, female dogs neutered before 1 year had a 20% risk of a joint disorder. Intact male and female dogs had 7% and 2% risks of having at least one cancer, respectively. Male dogs neutered before 1 year had a significantly elevated risk of cancer, 28%, whereas female dogs lacked any elevated risk. Intact female dogs had a 12% rate of PYO. The guideline is to neuter male dogs no earlier than 24 months of age due to joint disorders. With female dogs, it is suggested to neuter no earlier than 12 months of age due to a non-significant elevated incidence of cranial cruciate ligament tear and also due to the large body size.

### Newfoundland

Weighing over 100 pounds, this breed is currently ranked #42 (24) and, due to its love for water, has a history of assisting in maritime environments. The Newfoundland is non-aggressive and low in activity, but is average on demand for affection and is average on trainability, making for a very large dog somewhat easy to manage (25). The study population consisted of 74 intact male, 40 neutered male, 59 intact female, and 39 neutered female dogs, for a total sample of 212 cases. Intact male and female dogs had 10% and 7% rates of having at least one joint disorder, respectively. Males' rates after neutering were not significantly elevated, but females' rates for having a joint disorder were significantly elevated: 39% were affected when neutered in the 1^st^ year. There were no cancers in intact female dogs, and only a 3% rate for intact male dogs, with no significant elevations with neutering. Neutering female dogs before 1 year was associated with an 18% prevalence of UI, though not a significant increase over intact female dogs. Cases of MC and PYO were rarely recorded. The spaying guideline for female dogs is to neuter no earlier than 12 months of age. Despite no significant data against early neutering, it may be a cautious choice to neuter male dogs no earlier than 12 months of age due to the large body size of the breed.

### Rhodesian Ridgeback

Imported from South Africa, this breed is currently ranking #41 (24). Blended from the native Ridgeback and European breeds, the Ridgeback is well above average in aggression and somewhat above average in trainability, with low activity and affection (25). The study population consisted of 54 intact male, 69 neutered male, 30 intact female, and 56 neutered female dogs, for a total sample of 209 cases. Intact male and female dogs had 14% and 13% risk for at least one cancer, respectively. Neutering female dogs before 6 months provided the only significant increase in cancers, with a 25% rate of MCT compared with the 3% rate in intact female dogs. MC, PYO, and UI were rarely recorded in these female dogs. The guideline is to neuter no earlier than 6 months for male dogs and at least that for female dogs, given their cases of MCT with early neutering.

### Siberian Husky

This breed ranking #21 (24) is the most popular breed and fastest of the sled dogs. Historically, from the Siberian Chukchi people and then also the Alaskan Inuit culture, Huskies are quite high on aggressive traits and excessive barking and are only average for trainability (25). The study population consisted of 64 intact male, 77 neutered male, 48 intact female, and 77 neutered female dogs, from a total sample of 266 cases. Intact male and female dogs both had a 2% risk for joint disorders. Although neutering resulted in no significant risks for joint disorders over that of intact dogs, female dogs neutered before 1 year had a 12% chance of CCL, which would perhaps be significant with a larger dataset. Intact male and female dogs had 10% and 6% risks of at least one cancer, respectively, with no elevation associated with neutering. MC and PYO in intact female dogs were both 2%, but their prevalence in female dogs neutered 2–8 years were 9% and 4%, respectively. However, neither of these results was significant. The guideline is to neuter male dogs no earlier than 6 months of age. Given a trend for elevated CCL in early neutered female dogs, it is a cautious choice to neuter female dogs no earlier than 12 months of age.

## Discussion

This study extends the available information on five additional large breeds of dogs, showing their vulnerability to increased joint disorders and/or cancers with early neutering. As with results from previously studied breeds, the increased risks were specific for each breed. Regarding the mechanism of some increases in joint disorders with early neutering, larger dogs are known to have closure of the growth plates at higher ages than smaller dogs. Unfortunately, systematic data by breed on growth plate closure or puberty are not available. However, it was reported that the giant breeds, such as Great Danes, Mastiffs, or Saint Bernards, sometimes have closure at ages beyond the published ranges for dogs, reflecting their delays of closure of some long bones by the periods of up to 3–6 months beyond typical published data (26). The earlier closure in smaller dogs is generally accepted, and in one study, smaller dogs and cats were found to show earlier ranges of ages of closure (27). Another study of large and small breeds of dogs reported that large breeds had closure of their growth plates at a later chronological age (28).

This group of dogs showed a much lower incidence of hip and elbow disorders/abnormalities for intact Mastiff and Newfoundland dogs than OFA records of dogs being screened as breeders (percentage of HD followed by the percentage of ED): Mastiff: male, 2% and 2%–female, 6% and 4% vs. 21% and 16% of combined males and females from OFA; Newfoundland: male, 3% and 3%–female, 2% and 3% vs. 26% and 24% of combined male and female dogs from OFA. The higher OFA incidences of HD and ED in these two very large breeds may reflect more strict measures of abnormality than is symptomatic enough to lead to a diagnosis and also could be influenced by breed differences in geographic areas or by an elevated proportion of dog owners contacting the OFA due to the dog's known issues or expected issues with these disorders. Using data from 1954 to 2003, Witsberger et al. examined HD and CCL and reported high rates for male and female Newfoundland dogs: 17% and 9% (10). The same two disorders in the current dataset resulted in HD and CCL rates for dogs neutered in the 1^st^ year: male: 9% and 22%; female: 13% and 38%. The rates for intact dogs were as follows: male: 3% and 10%; female: 2% and 7%, suggesting that early neutering causes much of the very high rate for Newfoundlands. Male dogs neutered at 2–8 years had no joint disorders, but 18% of the female Newfoundland dogs had CCL.

The results with German Short/Wirehaired Pointers having increased risks for both joint disorders and cancers due to the age of neutering are somewhat unusual. Owners can be advised to delay neutering until 1 year and reduce these risks. With female Rhodesian Ridgebacks, the increased risk for MCT with very early neutering is easily avoided by waiting until the dog is 6 months old. The vulnerability of female Siberian Huskies to CCL with neutering before 1 year, while not significant, suggests that a cautious choice would be to wait until the dog is 1 year of age.

These decisions ultimately depend on the owners' preferences. The data can be complicated. As an example, the female Doberman has no increased risk for joint disorders or cancers if neutered beyond 12 months of age, as shown in Table 1. However, neutering between 1 and 2 years of age was associated with very elevated risks for UI−19% of female dogs.

Recovery from neutering is more problematic for older dogs than when still a puppy: perhaps creating a transient welfare challenge compared with neutering at younger ages. Neutering when the dog is older is also more expensive for the client. As mentioned earlier, increased fearfulness may be associated with neutering for some dogs (16, 17), a heightened risk for an ongoing issue with behavior and for the welfare of the dog. The situation has become increasingly complex when compared with the past practice of neutering most dogs early in life. Currently, dog owners are likely to be considering several factors when making their decisions on whether and when to neuter their dogs. Veterinarians can play an important educational role in providing information that relates to the clients' particular situations and objectives with their dogs.

These results offer information on the earliest ages to consider neutering without increasing risks for the mentioned joint disorders or cancers. Whether or not to neuter is a separate question for consideration by the handler consulting with the veterinarian. Some dog owners will prefer not to neuter the dog, and if reproduction is planned for the dog, neutering will at least be delayed. Humane societies placing dogs have complex considerations given the high risk for some dogs placed to be allowed to mate and some laws requiring neutering of dogs prior to placement. Alternative methods are increasing, and increasing numbers of clinicians are offering them. Perhaps some humane societies will begin offering options for preventing reproduction that does not involve gonadal hormone withdrawal, at least for large dogs.

## Limitations

Although a variety of alternative methods exist and are being developed to prevent the reproduction of male and female dogs, our data did not include information on these other methods. The results reported here are due to gonadal hormone withdrawal and/or increase in gonadotropic hormones; thus, it is likely that other methods of removing gonadal hormones would have similar results.

## Conclusions

Decisions on whether and when to neuter a specific dog are complicated and reflect a consideration of the dog's breed, health status, and living situation, as well as available information pertaining to risks for these joint disorders and cancers. These varied results highlight the complex effects of neutering and the age at which it is conducted. They point to the importance of personalized decision-making regarding the neutering of dogs, with consideration of relevant information available on the dog's breed and sex, as well as aspects of the dog's lifestyle.

## Data availability statement

The datasets presented in this study can be found in online repositories. The names of the repository/repositories and accession number(s) can be found below: Figshare, 10.6084/m9.figshare.24280054 – When accepted. Current private link — https://figshare.com/s/eaefceb41c81372cafbf.

## Author contributions

LH: Project administration, Writing – original draft, Supervision, Conceptualization. AT: Project administration, Writing – review & editing, Validation, Supervision, Formal analysis, Data curation. BH: Investigation, Conceptualization, Writing – review & editing. NW: Writing – review & editing, Software, Methodology, Formal analysis. ML: Writing – review & editing, Investigation. MB: Writing – review & editing, Investigation. JL: Writing – review & editing, Investigation. MH: Writing – review & editing, Investigation. SC: Writing – review & editing, Investigation. JC: Writing – review & editing.
