# Supplementary material for: Assisting decision-making on age of neutering for German Short/Wirehaired Pointer, Mastiff, Newfoundland, Rhodesian Ridgeback, Siberian Husky: associated joint disorders, cancers, and urinary incontinence
Source: Front Vet Sci. 2024 Apr 12;11:1322276. doi: 10.3389/fvets.2024.1322276 (PMC11046931; doi:10.3389/fvets.2024.1322276)
Supplement: Supplementary file 1 [file Data_Sheet_1.docx]

**Supplementary Material. APPENDIX 1**

**Dataset and Analyses for Each of Five Breeds**

**German Short/Wire-haired Pointer**

The study population was 150 intact males, 89 neutered males, 90 intact females, and 114 neutered females, for a total sample of 443 cases.

**Joint Disorders: hip dysplasia (HD), cranial cruciate ligament rupture (CCL), and elbow dysplasia (ED).** In this sample, 3% and 2% of males and females, respectively, were diagnosed with one or more joint disorders. Males neutered at 6-11 months had elevated CCL, and females neutered before six months had elevated joint disorders—38%.

**Cancers: lymphoma (LSA), mast cell tumor (MCT), hemangiosarcoma (HSA), and**

**osteosarcoma (OSA).** Neutering males before six months adversely affected MCT and HSA, and neutering females at 6-11 months increased LSA.

**Mammary Cancer (MC), Pyometra (PYO) and Urinary Incontinence (UI).** MC and PYO affected 4% of females. Females neutered before 12 months had 6% incidence of UI.

**Bottom Line.** Given the increased rates of both joint disorders and cancers for both males and females with early neutering, the suggested guideline is neutering no earlier than 12 months of age. This also reduces a female’s likelihood of UI.

**Joint Disorders.** For ages one through 11 years, for each neuter period, shown are number of cases over number in the pool, with percentages given in parentheses. When bolded the incidence is significantly above that of intact dogs. Bold values indicate significance over the intact group.

|  | **HD** | **CCL** | **ED** | **At Least One** |
| --- | --- | --- | --- | --- |
| Male < 6 months | 0/12 (0) | 0/12 (0) | 0/12 (0) | 0/12 (0) |
| Male 6 – 11 months | 0/19 (0) | **1/19 (5.26)** | 0/19 (0) | 1/19 (5.26) |
| (Male < 1 year) | 0/31 (0) | **1/31 (3.23)** | 0/31 (0) | 1/31 (3.23) |
| Male 1 year | 0/15 (0) | 0/15 (0) | 0/15 (0) | 0/15 (0) |
| Male 2 – 8 years | 0/33 (0) | 0/35 (0) | 0/35 (0) | 0/33 (0) |
| Male Intact | 5/151 (3.31) | 0/150 (0) | 0/150 (0) | 5/151 (3.31) |
|  |  |  |  |  |
| Female < 6 months | **1/13 (7.69)** | **4/13 (30.77)** | 0/13 (0) | **5/13 (38.46)** |
| Female 6 – 11 months | 0/25 (0) | 1/25 (4) | 0/25 (0) | 1/25 (4) |
| (Female < 1 year) | 1/38 (2.63) | **5/38 (13.16)** | 0/38 (0) | **6/38 (15.79)** |
| Female 1 year | 0/17 (0) | 0/17 (0) | 0/17 (0) | 0/17 (0) |
| Female 2 – 8 years | 0/50 (0) | 1/51 (1.96) | 0/52 (0) | 1/49 (2.04) |
| Female Intact | 1/91 (1.1) | 1/91 (1.1) | 0/90 (0) | 2/92 (2.17) |

**Cancers.** For ages one through 11 years, for each neuter period, shown are number of cases over number in the pool, with percentages given in parentheses. When bolded the incidence is significantly above that of intact dogs. Bold values indicate significance over the intact group.

|  | **LSA** | **MCT** | **HSA** | **OSA** | **At Least One** |
| --- | --- | --- | --- | --- | --- |
| Male < 6 months | 0/12 (0) | **1/12 (8.33)** | **1/12 (8.33)** | 0/12 (0) | 2/12 (16.67) |
| Male 6 – 11 months | 1/18 (5.56) | 0/19 (0) | 0/19 (0) | 0/19 (0) | 1/18 (5.56) |
| (Male < 1 year) | 1/30 (3.33) | 1/31 (3.23) | **1/31 (3.23)** | 0/31 (0) | 3/30 (10) |
| Male 1 year | 0/15 (0) | **1/15 (6.67)** | 0/15 (0) | 0/15 (0) | 1/15 (6.67) |
| Male 2 – 8 years | 1/35 (2.86) | 1/33 (3.03) | 1/35 (2.86) | 0/35 (0) | 3/33 (9.09) |
| Male Intact | 5/148 (3.38) | 3/151 (1.99) | 1/147 (0.68) | 0/150 (0) | 9/146 (6.16) |
|  |  |  |  |  |  |
| Female < 6 months | 1/13 (7.69) | 0/13 (0) | 0/13 (0) | 0/13 (0) | 1/13 (7.69) |
| Female 6 – 11 months | **3/24 (12.5)** | 0/25 (0) | 0/25 (0) | 0/25 (0) | **3/24 (12.5)** |
| (Female < 1 year) | **4/37 (10.81)** | 0/38 (0) | 0/38 (0) | 0/38 (0) | **4/37 (10.81)** |
| Female 1 year | 0/17 (0) | 0/17 (0) | 0/17 (0) | 0/17 (0) | 0/17 (0) |
| Female 2 – 8 years | 0/52 (0) | 1/51 (1.96) | 0/52 (0) | 0/52 (0) | 1/51 (1.96) |
| Female Intact | 0/90 (0) | 1/90 (1.11) | 0/89 (0) | 0/90 (0) | 1/89 (1.12) |

**Mammary Cancer, Pyometra, and Urinary Incontinence in Females.** For ages one through 11 years, for each neuter period, shown are number of cases over number in the pool, with percentages given in parentheses. When bolded the incidence is significantly above that of intact dogs.

|  | **MC** | **PYO** | **UI** |
| --- | --- | --- | --- |
| Female < 6 months | 0/13 (0) | 0/13 (0) | 1/11 (9.09) |
| Female 6 – 11 months | 0/25 (0) | 0/25 (0) | 1/21 (4.76) |
| (Female < 1 year) | 0/38 (0) | 0/38 (0) | 2/32 (6.25) |
| Female 1 year | 0/17 (0) | 0/17 (0) | 0/16 (0) |
| Female 2 – 8 years | 0/47 (0) | 0/47 (0) | 1/50 (2) |
| Female Intact | 4/92 (4.35) | 4/92 (4.35) | 0/90 (0) |

**Urinary Incontinence in Males**. For ages one through 11 years, for each neuter period, shown are number of cases over number in the pool, with percentages given in parentheses. When bolded the incidence is significantly above that of intact dogs.

|  | **UI** |
| --- | --- |
| Male < 6 months | 0/12 (0) |
| Male 6 – 11 months | 0/19 (0) |
| (Male < 1 year) | 0/31 (0) |
| Male 1 year | 0/15 (0) |
| Male 2 – 8 years | 1/35 (2.86) |
| Male Intact | 0/150 (0) |

**Mastiff**

The study population was 148 intact males, 61 neutered males, 70 intact females, and 82 neutered females, for a total sample of 361 cases.

**Joint Disorders: hip dysplasia (HD), cranial cruciate ligament rupture (CCL), and elbow dysplasia (ED).** In this sample, 6% and 9% of intact males and females, respectively, had at least one joint disorder. With neutering at 6-11 months, the males’ rate of having a joint disorder was a significant 33%, due to CCL. Neutering while one year had a significant 15% risk. Females neutered in the first 12 months had an elevated but non-significant 20% rate.

**Cancers: lymphoma (LSA), mast cell tumor (MCT), hemangiosarcoma (HSA), and**

**osteosarcoma (OSA).** The rate of having one cancer was 7% and 2% for intact males and females, respectively. Males neutered before one year had a significant 28% risk of having a cancer. Neutering before one year for females carried a non-significant 9% risk.

**Mammary Cancer (MC), Pyometra (PYO) and Urinary Incontinence (UI).** Intact females had a 12% rate of PYO. In other regards, females and males had extremely few or no cases.

**Bottom Line.** The guideline is to neuter males no earlier than 24 months of age due to joint disorders, and neutering in the first year also would elevate cancer risk. With females, it is suggested to neuter no earlier than 12 months of age due to trends for elevated cranial cruciate ligament tear and cancer risks, and also due to the large body size.

**Joint Disorders.** For ages one through 11 years, for each neuter period, shown are number of cases over number in the pool, with percentages given in parentheses. When bolded the incidence is significantly above that of intact dogs. Bold values indicate significance over the intact group.

|  | **HD** | **CCL** | **ED** | **At Least One** |
| --- | --- | --- | --- | --- |
| Male < 6 months | 0/7 (0) | 0/7 (0) | 0/7 (0) | 0/7 (0) |
| Male 6 – 11 months | 0/12 (0) | **4/12 (33.33)** | 0/12 (0) | **4/12 (33.33)** |
| (Male < 1 year) | 0/19 (0) | **4/19 (21.05)** | 0/19 (0) | **4/19 (21.05)** |
| Male 1 year | 1/20 (5) | **2/20 (10)** | 0/20 (0) | **3/20 (15)** |
| Male 2 – 8 years | 0/19 (0) | 0/17 (0) | 0/20 (0) | 0/17 (0) |
| Male Intact | 3/147 (2.04) | 6/144 (4.17) | 3/144 (2.08) | 8/144 (5.56) |
|  |  |  |  |  |
| Female < 6 months | 1/10 (10) | 2/10 (20) | 0/10 (0) | 2/10 (20) |
| Female 6 – 11 months | 0/21 (0) | 4/21 (19.05) | 0/21 (0) | 4/20 (20) |
| (Female < 1 year) | 1/31 (3.23) | 6/31 (19.35) | 0/31 (0) | 6/30 (20) |
| Female 1 year | 0/9 (0) | 0/10 (0) | 0/10 (0) | 0/9 (0) |
| Female 2 – 8 years | 0/38 (0) | 3/36 (8.33) | 0/39 (0) | 2/34 (5.88) |
| Female Intact | 4/71 (5.63) | 4/69 (5.8) | 3/70 (4.29) | 6/69 (8.7) |

**Cancers.** For ages one through 11 years, for each neuter period, shown are number of cases over number in the pool, with percentages given in parentheses. When bolded the incidence is significantly above that of intact dogs. Bold values indicate significance over the intact group.

|  | **LSA** | **MCT** | **HSA** | **OSA** | **At Least One** |
| --- | --- | --- | --- | --- | --- |
| Male < 6 months | **1/7 (14.29)** | 0/7 (0) | 0/7 (0) | 0/7 (0) | 1/7 (14.29) |
| Male 6 – 11 months | 1/12 (8.33) | 3/12 (25) | 0/12 (0) | 0/11 (0) | 4/11 (36.36) |
| (Male < 1 year) | **2/19 (10.53)** | 3/19 (15.79) | 0/19 (0) | 0/18 (0) | **5/18 (27.78)** |
| Male 1 year | 0/20 (0) | 0/20 (0) | 0/19 (0) | 0/20 (0) | 0/19 (0) |
| Male 2 – 8 years | 1/20 (5) | 1/19 (5.26) | 0/20 (0) | 0/20 (0) | 2/19 (10.53) |
| Male Intact | 2/147 (1.36) | 3/149 (2.01) | 2/147 (1.36) | 4/144 (2.8) | 10/143 (6.99) |
|  |  |  |  |  |  |
| Female < 6 months | 1/10 (10) | 1/10 (10) | 0/10 (0) | 0/10 (0) | 2/10 (20) |
| Female 6 – 11 months | 0/22 (0) | 0/22 (0) | 1/22 (4.55) | 0/22 (0) | 1/22 (4.55) |
| (Female < 1 year) | 1/32 (3.13) | 1/32 (3.13) | 1/32 (3.13) | 0/32 (0) | 3/32 (9.38) |
| Female 1 year | 0/10 (0) | 0/10 (0) | 0/10 (0) | 0/10 (0) | 0/10 (0) |
| Female 2 – 8 years | 1/39 (2.56) | 2/39 (5.13) | 1/39 (2.56) | 1/38 (2.63) | 5/38 (13.16) |
| Female Intact | 0/67 (0) | 0/70 (0) | 0/69 (0) | 1/69 (1.45) | 1/66 (1.52) |

**Mammary Cancer, Pyometra, and Urinary Incontinence in Females.** For ages one through 11 years, for each neuter period, shown are number of cases over number in the pool, with percentages given in parentheses. When bolded the incidence is significantly above that of intact dogs.

|  | **MC** | **PYO** | **UI** |
| --- | --- | --- | --- |
| Female < 6 months | 0/10 (0) | 0/10 (0) | 0/7 (0) |
| Female 6 – 11 months | 0/22 (0) | 0/22 (0) | 0/21 (0) |
| (Female < 1 year) | 0/32 (0) | 0/32 (0) | 0/28 (0) |
| Female 1 year | 0/10 (0) | 0/10 (0) | 0/8 (0) |
| Female 2 – 8 years | 0/39 (0) | 0/33 (0) | 1/33 (3.03) |
| Female Intact | 0/70 (0) | 9/75 (12) | 0/70 (0) |

**Urinary Incontinence in Males**. For ages one through 11 years, for each neuter period, shown are number of cases over number in the pool, with percentages given in parentheses. When bolded the incidence is significantly above that of intact dogs.

|  | **UI** |
| --- | --- |
| Male < 6 months | 0/7 (0) |
| Male 6 – 11 months | 0/12 (0) |
| (Male < 1 year) | 0/19 (0) |
| Male 1 year | 0/19 (0) |
| Male 2 – 8 years | 0/18 (0) |
| Male Intact | 0/147 (0) |

**Newfoundland**

The study population was 74 intact males, 40 neutered males, 59 intact females, and 39 neutered females, for a total sample of 212 cases.

**Joint disorders: hip dysplasia (HD), cranial cruciate ligament rupture (CCL), and elbow dysplasia (ED).** Intact males and females had 10% and 7% rates of having at least one joint disorder, respectively. Males’ rates after neutering were not significantly elevated, but females’ rates for having a joint disorder were significantly elevated, 39% were affected when neutered in the first year.

**Cancers: lymphoma (LSA), mast cell tumor (MCT), hemangiosarcoma (HSA), and osteosarcoma (OSA).** There were no cancers in intact females, and only a 3% rate for intact males, with no significant elevations with neutering. Although insignificant, two males neutered at 2-8 years had cancers, resulting in a 15% rate.

**Mammary Cancer (MC), Pyometra (PYO) and Urinary Incontinence (UI).** Neutering females before one year was associated with an 18% rate of UI. Although not a significant increase over intact females, it merits watching. Cases of MC and PYO were rarely recorded.

**Bottom line:** The guideline for females is to neuter no earlier than 12 months of age. Despite no significant data against early neutering, it may be a cautious choice to neuter males no earlier than 12 months of age due to the large body size of the breed.

**Joint Disorders.** For ages one through 11 years, for each neuter period, shown are number of cases over number in the pool, with percentages given in parentheses. When bolded the incidence is significantly above that of intact dogs. Bold values indicate significance over the intact group.

|  | **HD** | **CCL** | **ED** | **At Least One** |
| --- | --- | --- | --- | --- |
| Male < 6 months | 0/3 (0) | 1/2 (50) | 0/2 (0) | 1/2 (50) |
| Male 6 – 11 months | 1/8 (12.5) | 1/7 (14.29) | 0/8 (0) | 1/7 (14.29) |
| (Male < 1 year) | 1/11 (9.09) | 2/9 (22.22) | 0/10 (0) | 2/9 (22.22) |
| Male 1 year | 1/14 (7.14) | 2/10 (20) | 0/10 (0) | 3/10 (30) |
| Male 2 – 8 years | 0/11 (0) | 0/11 (0) | 0/12 (0) | 0/10 (0) |
| Male Intact | 2/71 (2.82) | 7/69 (10.14) | 2/69 (2.9) | 7/67 (10.45) |
|  |  |  |  |  |
| Female < 6 months | 0/5 (0) | **2/5 (40)** | **1/5 (20)** | **2/5 (40)** |
| Female 6 – 11 months | **2/10 (20)** | **3/8 (37.5)** | **1/11 (9.09)** | **3/8 (37.5)** |
| (Female < 1 year) | **2/15 (13.33)** | **5/13 (38.46)** | **2/16 (12.5)** | **5/13 (38.46)** |
| Female 1 year | 0/6 (0) | 0/6 (0) | 0/6 (0) | 0/6 (0) |
| Female 2 – 8 years | 0/13 (0) | 2/11 (18.18) | 0/12 (0) | 2/11 (18.18) |
| Female Intact | 1/59 (1.69) | 4/61 (6.56) | 2/60 (3.33) | 4/60 (6.67) |

**Cancers.** For ages one through 11 years, for each neuter period, shown are number of cases over number in the pool, with percentages given in parentheses. When bolded the incidence is significantly above that of intact dogs.

|  | **LSA** | **MCT** | **HSA** | **OSA** | **At Least One** |
| --- | --- | --- | --- | --- | --- |
| Male < 6 months | 0/3 (0) | 0/3 (0) | 0/3 (0) | 0/3 (0) | 0/3 (0) |
| Male 6 – 11 months | 1/8 (12.5) | 0/8 (0) | 0/8 (0) | 1/8 (12.5) | 2/8 (25) |
| (Male < 1 year) | 1/11 (9.09) | 0/11 (0) | 0/11 (0) | 1/11 (9.09) | 2/11 (18.18) |
| Male 1 year | 1/13 (7.69) | 0/14 (0) | 0/14 (0) | 0/14 (0) | 1/13 (7.69) |
| Male 2 – 8 years | 0/13 (0) | 0/14 (0) | 1/14 (7.14) | 1/14 (7.14) | 2/13 (15.38) |
| Male Intact | 2/74 (2.7) | 0/74 (0) | 0/73 (0) | 0/73 (0) | 2/72 (2.78) |
|  |  |  |  |  |  |
| Female < 6 months | 0/6 (0) | 0/6 (0) | 0/6 (0) | 0/6 (0) | 0/6 (0) |
| Female 6 – 11 months | 0/11 (0) | 0/11 (0) | 0/11 (0) | 1/10 (10) | 1/10 (10) |
| (Female < 1 year) | 0/17 (0) | 0/17 (0) | 0/17 (0) | 1/16 (6.25) | 1/16 (6.25) |
| Female 1 year | 0/6 (0) | 0/7 (0) | 0/7 (0) | 0/7 (0) | 0/6 (0) |
| Female 2 – 8 years | 0/14 (0) | 0/14 (0) | 0/14 (0) | 0/14 (0) | 0/14 (0) |
| Female Intact | 0/59 (0) | 0/59 (0) | 0/59 (0) | 0/59 (0) | 0/59 (0) |

**Mammary Cancer (MC), Pyometra (PYO) and Urinary Incontinence (UI) in Females.** For ages one through 11 years, for each neuter period, hown are number of cases over number in the pool, with percentages given in parentheses. When bolded the incidence is significantly above that of intact dogs.

|  | **MC** | **PYO** | **UI** |
| --- | --- | --- | --- |
| Female < 6 months | 0/6 (0) | 0/6 (0) | 0/2 (0) |
| Female 6 – 11 months | 0/11 (0) | 0/11 (0) | 2/9 (22.22) |
| (Female < 1 year) | 0/17 (0) | 0/17 (0) | 2/11 (18.18) |
| Female 1 year | 0/7 (0) | 0/7 (0) | 0/6 (0) |
| Female 2 – 8 years | 0/14 (0) | 0/13 (0) | 1/14 (7.14) |
| Female Intact | 0/59 (0) | 1/60 (1.67) | 0/57 (0) |

Newfoundland males, urinary incontinence. For ages one through 11 years, for each neuter period, shown are number of cases over number in the pool, with percentages given in parentheses. When bolded the incidence is significantly above that of intact dogs.

|  | **UI** |
| --- | --- |
| Male < 6 months | 0/3 (0) |
| Male 6 – 11 months | 0/7 (0) |
| (Male < 1 year) | 0/10 (0) |
| Male 1 year | 0/13 (0) |
| Male 2 – 8 years | 0/14 (0) |
| Male Intact | 0/74 (0) |

**Rhodesian Ridgeback**

The study population was 54 intact males, 69 neutered males, 30 intact females, and 56 neutered females, for a total sample of 209 cases.

**Joint disorders: hip dysplasia (HD), cranial cruciate ligament rupture (CCL), and elbow dysplasia (ED).** Intact males and females had 2% and 3% risks of at least one joint disorder, respectively; no significant risk for a joint disorder was associated with neutering.

**Cancers: lymphoma (LSA), mast cell tumor (MCT), hemangiosarcoma (HSA), and**

**osteosarcoma (OSA).** Neutering females before 6 months provided the only significant increase in cancers, with a 25% rate of MCT over the 3% rate of MCT in intact females.

**Mammary Cancer (MC), Pyometra (PYO) and Urinary Incontinence (UI)**. MC, PYO, and UI were rarely recorded in these females.

**Bottom line.** The guideline is to neuter no earlier than six months for males and at least that for females, given their cases of MCT with early neutering.

**Joint Disorders.** For ages one through 11 years, for each neuter period, shown are number of cases over number in the pool, with percentages given in parentheses. When bolded the incidence is significantly above that of intact dogs.

|  | **HD** | **CCL** | **ED** | **At Least One** |
| --- | --- | --- | --- | --- |
| Male < 6 months | 0/10 (0) | 0/10 (0) | 0/9 (0) | 0/9 (0) |
| Male 6 – 11 months | 0/20 (0) | 0/20 (0) | 0/20 (0) | 0/20 (0) |
| (Male < 1 year) | 0/30 (0) | 0/30 (0) | 0/29 (0) | 0/29 (0) |
| Male 1 year | 0/11 (0) | 0/11 (0) | 0/11 (0) | 0/11 (0) |
| Male 2 – 8 years | 0/26 (0) | 0/27 (0) | 0/27 (0) | 0/26 (0) |
| Male Intact | 1/54 (1.85) | 0/54 (0) | 1/54 (1.85) | 1/54 (1.85) |
|  |  |  |  |  |
| Female < 6 months | 0/7 (0) | 0/8 (0) | 0/8 (0) | 0/7 (0) |
| Female 6 – 11 months | 0/18 (0) | 0/18 (0) | 0/18 (0) | 0/18 (0) |
| (Female < 1 year) | 0/25 (0) | 0/26 (0) | 0/26 (0) | 0/25 (0) |
| Female 1 year | 0/9 (0) | 0/9 (0) | 0/9 (0) | 0/9 (0) |
| Female 2 – 8 years | 0/20 (0) | 1/20 (5) | 0/20 (0) | 1/20 (5) |
| Female Intact | 0/30 (0) | 1/30 (3.33) | 0/29 (0) | 1/29 (3.45) |

**Cancers.** For ages one through 11 years, for each neuter period, shown are number of cases over number in the pool, with percentages given in parentheses. When bolded the incidence is significantly above that of intact dogs. Bold values indicate significance over the intact group.

|  | **LSA** | **MCT** | **HSA** | **OSA** | **At Least One** |
| --- | --- | --- | --- | --- | --- |
| Male < 6 months | 0/10 (0) | 0/10 (0) | 0/10 (0) | 0/9 (0) | 0/9 (0) |
| Male 6 – 11 months | 1/20 (5) | 0/19 (0) | 0/20 (0) | 0/20 (0) | 1/19 (5.26) |
| (Male < 1 year) | 1/30 (3.33) | 0/29 (0) | 0/30 (0) | 0/29 (0) | 1/28 (3.57) |
| Male 1 year | 0/11 (0) | 0/11 (0) | 0/11 (0) | 0/11 (0) | 0/11 (0) |
| Male 2 – 8 years | 0/26 (0) | 3/24 (12.5) | 0/27 (0) | 0/26 (0) | 3/22 (13.64) |
| Male Intact | 3/55 (5.45) | 3/56 (5.36) | 0/54 (0) | 3/55 (5.45) | 8/58 (13.79) |
|  |  |  |  |  |  |
| Female < 6 months | 0/8 (0) | **2/8 (25)** | 0/8 (0) | 1/8 (12.5) | 2/8 (25) |
| Female 6 – 11 months | 0/17 (0) | 0/18 (0) | 0/17 (0) | 0/18 (0) | 0/17 (0) |
| (Female < 1 year) | 0/25 (0) | 2/26 (7.69) | 0/25 (0) | 1/26 (3.85) | 2/25 (8) |
| Female 1 year | 0/9 (0) | 0/8 (0) | 0/9 (0) | 0/9 (0) | 0/8 (0) |
| Female 2 – 8 years | 1/20 (5) | 2/19 (10.53) | 0/20 (0) | 0/20 (0) | 3/19 (15.79) |
| Female Intact | 3/30 (10) | 1/31 (3.23) | 0/30 (0) | 0/29 (0) | 4/30 (13.33) |

**Mammary Cancer (MC), Pyometra (PYO) and Urinary Incontinence (UI) in Females**. For ages one through 11 years, for each neuter period, shown are number of cases over number in the pool, with percentages given in parentheses. When bolded the incidence is significantly above that of intact dogs.

|  | **MC** | **PYO** | **UI** |
| --- | --- | --- | --- |
| Female < 6 months | 0/8 (0) | 0/8 (0) | 0/6 (0) |
| Female 6 – 11 months | 0/18 (0) | 0/18 (0) | 0/15 (0) |
| (Female < 1 year) | 0/26 (0) | 0/26 (0) | 0/21 (0) |
| Female 1 year | 0/9 (0) | 0/9 (0) | 0/9 (0) |
| Female 2 – 8 years | 0/19 (0) | 0/20 (0) | 0/17 (0) |
| Female Intact | 1/31 (3.23) | 0/30 (0) | 0/29 (0) |

**Urinary Incontinence in Males**. For ages one through 11 years, for each neuter period, the occurrence of urinary incontinence (UI) is portrayed. Shown are number of cases over number in the pool, with percentages given in parentheses. When bolded the incidence is significantly above that of intact dogs.

|  | **UI** |
| --- | --- |
| Male < 6 months | 0/10 (0) |
| Male 6 – 11 months | 1/19 (5.26) |
| (Male < 1 year) | 1/29 (3.45) |
| Male 1 year | 0/10 (0) |
| Male 2 – 8 years | 0/23 (0) |
| Male Intact | 0/54 (0) |

**Siberian Husky**

The study population was 64 intact males, 77 neutered males, 48 intact females, and 77 neutered females, for a total sample of 266 cases.

**Joint disorders: hip dysplasia (HD), cranial cruciate ligament rupture (CCL), and elbow dysplasia (ED).** Intact males and females both had 2% chance of a joint disorder. Although neutering resulted in no significant risks for joint disorders over that of intact dogs, females neutered before a year had a 12% chance of CCL; this could be significant with a larger dataset.

**Cancers: lymphoma (LSA), mast cell tumor (MCT), hemangiosarcoma (HSA), and**

**osteosarcoma (OSA).** Intact males and females had 10% and 6% risks of at least one cancer, respectively, with no elevation associated with neutering.

**Mammary Cancer (MC), Pyometra (PYO) and Urinary Incontinence (UI)**. MC and PYO in intact females were both 2%, but rates in females neutered 2-8 years were 9% and 4% respectively. However, neither of these results were significant.

**Bottom line.** The guideline is to neuter males no earlier than six months of age. Given a trend for elevated CCL in early neutered females, it is a cautious choice to neuter females no earlier than 12 months of age.

**Joint disorders.** For ages one through 11 years, for each neuter period, shown are number of cases over number in the pool, with percentages given in parentheses. When bolded the incidence is significantly above that of intact dogs.

|  | **HD** | **CCL** | **ED** | **At Least One** |
| --- | --- | --- | --- | --- |
| Male < 6 months | 0/16 (0) | 0/16 (0) | 0/15 (0) | 0/15 (0) |
| Male 6 – 11 months | 0/17 (0) | 1/17 (5.88) | 0/18 (0) | 1/17 (5.88) |
| (Male < 1 year) | 0/33 (0) | 1/33 (3.03) | 0/33 (0) | 1/32 (3.13) |
| Male 1 year | 0/17 (0) | 0/17 (0) | 0/17 (0) | 0/17 (0) |
| Male 2 – 8 years | 0/23 (0) | 0/24 (0) | 0/24 (0) | 0/23 (0) |
| Male Intact | 0/64 (0) | 1/63 (1.59) | 0/64 (0) | 1/63 (1.59) |
|  |  |  |  |  |
| Female < 6 months | 0/12 (0) | 1/12 (8.33) | 0/12 (0) | 1/12 (8.33) |
| Female 6 – 11 months | 0/22 (0) | 3/22 (13.64) | 0/22 (0) | 3/22 (13.64) |
| (Female < 1 year) | 0/34 (0) | 4/34 (11.76) | 0/34 (0) | 4/34 (11.76) |
| Female 1 year | 0/14 (0) | 0/14 (0) | 0/14 (0) | 0/14 (0) |
| Female 2 – 8 years | 0/24 (0) | 1/24 (4.17) | 0/24 (0) | 1/24 (4.17) |
| Female Intact | 1/48 (2.08) | 0/48 (0) | 0/48 (0) | 1/48 (2.08) |

**Cancers.** For ages one through 11 years, for each neuter period, shown are number of cases over number in the pool, with percentages given in parentheses. When bolded the incidence is significantly above that of intact dogs.

|  | **LSA** | **MCT** | **HSA** | **OSA** | **At Least One** |
| --- | --- | --- | --- | --- | --- |
| Male < 6 months | 0/16 (0) | 0/16 (0) | 0/16 (0) | 0/16 (0) | 0/16 (0) |
| Male 6 – 11 months | 0/18 (0) | 0/18 (0) | 0/18 (0) | 0/18 (0) | 0/18 (0) |
| (Male < 1 year) | 0/34 (0) | 0/34 (0) | 0/34 (0) | 0/34 (0) | 0/34 (0) |
| Male 1 year | 0/16 (0) | 0/17 (0) | 0/17 (0) | 0/17 (0) | 0/16 (0) |
| Male 2 – 8 years | 1/23 (4.35) | 0/24 (0) | 0/24 (0) | 0/24 (0) | 1/23 (4.35) |
| Male Intact | 3/63 (4.76) | 1/64 (1.56) | 2/64 (3.13) | 0/63 (0) | 6/62 (9.68) |
|  |  |  |  |  |  |
| Female < 6 months | 0/12 (0) | 0/12 (0) | 0/12 (0) | 0/12 (0) | 0/12 (0) |
| Female 6 – 11 months | 0/22 (0) | 0/22 (0) | 0/21 (0) | 0/22 (0) | 0/21 (0) |
| (Female < 1 year) | 0/34 (0) | 0/34 (0) | 0/33 (0) | 0/34 (0) | 0/33 (0) |
| Female 1 year | 0/14 (0) | 0/14 (0) | 0/14 (0) | 0/14 (0) | 0/14 (0) |
| Female 2 – 8 years | 0/23 (0) | 0/23 (0) | 0/24 (0) | 1/24 (4.17) | 1/22 (4.55) |
| Female Intact | 2/47 (4.26) | 1/49 (2.04) | 0/48 (0) | 0/48 (0) | 3/48 (6.25) |

**Mammary Cancer (MC), Pyometra (PYO) and Urinary Incontinence (UI) in Females**. For ages one through 11 years, for each neuter period, shown are number of female cases over number in the pool, with percentages given in parentheses. When bolded the incidence is significantly above that of intact dogs.

|  | **MC** | **PYO** | **UI** |
| --- | --- | --- | --- |
| Female < 6 months | 0/12 (0) | 0/12 (0) | 0/11 (0) |
| Female 6 – 11 months | 0/22 (0) | 0/22 (0) | 0/19 (0) |
| (Female < 1 year) | 0/34 (0) | 0/34 (0) | 0/30 (0) |
| Female 1 year | 0/14 (0) | 0/14 (0) | 1/14 (7.14) |
| Female 2 – 8 years | 2/23 (8.7) | 1/24 (4.17) | 1/22 (4.55) |
| Female Intact | 1/49 (2.04) | 1/47 (2.13) | 0/45 (0) |

**Urinary Incontinence in Males**. For ages one through 11 years, for each neuter period, the occurrence of urinary incontinence (UI) is portrayed. Shown are number of cases over number in the pool, with percentages given in parentheses. When bolded the incidence is significantly above that of intact dogs. Bold values indicate significance over the intact group.

|  | **UI** |
| --- | --- |
| Male < 6 months | 0/15 (0) |
| Male 6 – 11 months | 0/18 (0) |
| (Male < 1 year) | 0/33 (0) |
| Male 1 year | **1/16 (6.25)** |
| Male 2 – 8 years | 0/24 (0) |
